# Genetic Association Analysis of Paratuberculosis Forms in Holstein-Friesian Cattle

**DOI:** 10.1155/2014/321327

**Published:** 2014-05-20

**Authors:** Patricia Vázquez, Otsanda Ruiz-Larrañaga, Joseba M. Garrido, Mikel Iriondo, Carmen Manzano, Mikel Agirre, Andone Estonba, Ramón A. Juste

**Affiliations:** ^1^Department of Animal Health, NEIKER-Tecnalia, Berreaga 1, 48160 Derio, Bizkaia, Spain; ^2^Department of Genetics, Physical Anthropology and Animal Physiology, Faculty of Science and Technology, University of the Basque Country (UPV/EHU), Barrio Sarriena S/N, 48940 Leioa, Bizkaia, Spain

## Abstract

A genetic susceptibility to *Mycobacterium avium* subsp. *paratuberculosis* (MAP) infections in ruminants has been longtime suspected to exist. Recently, natural infections in cattle have been reclassified into *latent* and *patent* forms based on histopathological findings and their associations with immunological and microbiological variables. This study aims to explore whether these newly defined phenotypes are associated with twenty-four single-nucleotide polymorphisms (SNPs) in six bovine candidate genes: *nucleotide-binding oligomerization domain 2 (NOD2), solute carrier family 11 member A1 (SLC11A1), nuclear body protein SP110 (SP110), toll-like receptors (TLRs) 2* and *4*, and *CD209* (also known as *DC-SIGN, dendritic cell-specific ICAM3-grabbing nonintegrin*). SNPs were genotyped for 772 Holstein-Friesian animals (52.6% *apparently free*; 38.1% *latent*; 9.3% *patent*) by TaqMan OpenArray technology. Genotypic-phenotypic associations were assessed by logistic regression analysis adjusted for age at slaughter, under five models (codominant, dominant, recessive, overdominant, and log-additive), and corrected for multiple testing. The rs208222804 C allele (*CD209* gene) was found to be associated with *latent* paratuberculosis (log-additive model: *P* < 0.0034 after permutation procedure; OR = 0.64, 95% CI = 0.48–0.86). No significant association was detected between any SNP and the *patent* phenotype. Consequently, *CD209* gene may play a key role in the pathogenesis of bovine paratuberculosis.

## 1. Introduction

Paratuberculosis (PTB) is a worldwide mycobacterial infectious disease affecting domestic and wild ruminants [1] and resulting in large economic loses, particularly in dairy herds [2]. In most cases,* Mycobacterium avium* subsp.* paratuberculosis *(MAP) infects cattle subclinically and evidences of typical clinical signs (diarrhea, decreased milk productions, and weight loss) are normally limited to a low proportion of infected individuals aged two years or more [3].

Genetic susceptibility to MAP infection has been investigated in cattle, sheep, and deer [4]. Previous case-control studies in cattle have concluded diverse associations between genetic variants in innate immunity-related genes and PTB status, which made marker-assisted selection (MAS) a promising tool to improve PTB control. Among those susceptibility* loci*, some located in the* nucleotide-binding oligomerization domain 2 (NOD2), solute carrier family 11 member A1 (SLC11A1), nuclear body protein SP110 (SP110), *and* toll-like receptors (TLRs) 2 *and* 4 *genes have been previously identified by our group in a Spanish Holstein-Friesian population [5–8]. Additionally, we have recently found five SNPs in* CD209* (also known as* DC-SIGN, dendritic cell-specific ICAM3-grabbing nonintegrin*) showing epistatic interactions with* TLR2 *and* TLR4* genes among MAP infected cattle, which would support the implication of this gene on mycobacterial recognition and the onset of innate and adaptive immune responses against it [9, 10]. Furthermore, several studies based on either candidate genes or genome-wide association studies (GWAS) can be found in the literature, suggesting a large number of putative susceptibility [11–20] and tolerance [21]* loci* for MAP infection.

However, discrepancies between studies have been observed which make them unsuitable for direct application in MAS schemes for Holstein-Friesian breeding. This could be in large part due to the difficulties for estimating the true PTB status when using* in vivo* diagnostic testing, mainly ELISA and fecal culture/PCR, which are relatively insensitive tests compared with histopathological examination [22, 23]. These influences would lead to misclassified phenotypes and thus biased analyses [24], particularly in subclinical forms of infection.

In addition, it appears interesting to explore not only those MAP infected animals, traditionally defined as cases when positive to one or more diagnostic tests, but also those subgroups showing particular immunopathological features, with a special focus on the pathological variables proposed by González et al. [22]. To achieve this purpose, the recent approach to classifying MAP infection forms into “*latent*” and “*patent*” could prove to be very useful, since its hallmark is based on the relationship between the histopathological findings, the presence of specific antibodies against MAP, and the presence of viable forms or DNA of MAP in tissues [25]. This pathological criterion is what precisely marks the difference with other PTB classifications which are mainly based on microbiological variables and especially with MAP fecal shedding, such as those proposed by Nielsen and Toft [26] and Zanella et al. [21]. Hence, according to our simplified model, “*latent*” infections, corresponding to the* focal* immunopathological form of Pérez et al. [27] and González et al. [22] and defined by delimited granulomatous lesions and scarce humoral response or MAP presence, might represent forms of resistance that could prevent animals from developing more severe forms. In turn, “*patent*” infections, corresponding to* multifocal* and* diffuse* types of enteritis [22, 27] where there are an increased antibody production, substantial mycobacterial load, and high MAP viability rate, are much less prevalent but imply a higher infectious risk and a shortening of life expectancy [28]. Although not fully demonstrated, each of these pathological patterns of PTB seems to correspond to two differentiated pathways under MAP exposure. One is persisting or even increasing along with age, and the other one nearly disappeared after clinical disease has caused the death of affected individuals in their first years of life.

In this context, the aim of this study is to evaluate whether chronic intestinal inflammation and/or mycobacterial infection pathogenesis occurring in* latent *and* patent* forms of bovine PTB are genetically conditioned by SNP mutations of* NOD2, SLC11A1, SP110, TLR2, TLR4, *and* CD209* innate immunity genes, all previously reported to be associated with MAP infection.

## 2. Material and Methods

### 2.1. Animals and Sampling

Seven hundred and seventy-two Holstein-Friesian animals from eight Northeast Spain areas (Basque Country (44.2%), Catalonia (23.8%), Navarre (18.7), Cantabria (5.6%), Aragon (3.9%), Castile and León (2.3%), La Rioja (1.0%), and Asturias (0.5%)) were examined. For these regions, the estimates of PTB prevalence ranged from 50.0% to 70.0%, by using the parallel evaluation of immunological, microbiological, and histopathological results (*n* = 772). Animals were 5.6 years old on average. Except for three animals, which were aged between 20.2 and 23.1 months, the rest were adults (2 years or older).

A systematic blood and tissue sampling was weekly performed in two local abattoirs located in the Basque Country (Bilbao and Donostia-San Sebastián), from March 2007 to May 2010. In each sampling day, the average number of animals selected for the study varied from 4 to 6. Animals were chosen according to breed and age requirements, following the slaughter line order fixed by the slaughterhouse managers. Adult cattle were chosen because the chances of being exposed to MAP were higher than those for younger animals. Briefly, immediately after stunning and before bleeding, duplicate jugular venous whole blood samples were collected into 10 mL Vacutainer EDTA tubes (BD, Franklin Lakes, USA) to later perform the immunological and SNPs genotyping processes. Next, the gut package of each animal was identified and picked up. Macroscopic examination and selection of tissue samples for subsequent microbiological and histopathological studies were performed in NEIKER-Tecnalia necropsy room. More details on animal selection and methodological procedures for postmortem sample collection have been previously described [23, 25, 28].

Operations in both municipally owned companies were authorized by slaughterhouse management and carried out under the supervision of official veterinarians and complied with the pertinent legislations for safeguarding animal welfare (Basque Government Decree 454/1994, Spanish Government Law 32/2007 and Royal decree 731/2007, and European Council Regulation (EC) Number 1099/2009).

The date of birth of animals, as recorded in the EU bovine identification documents (Council Regulation (EC) Number 1760/2000), was provided by the slaughterhouse veterinary inspectors.

### 2.2. Determination of MAP Infection Status

For each animal, the infection status was investigated by serological, microbiological, and histopathological methods. Serum samples were evaluated for specific antibody production against MAP by using the two-step indirect Pourquier ELISA paratuberculosis kit (Institut Pourquier, Montpellier, France), currently IDEXX Paratuberculosis Screening and Verification Ab Tests (IDEXX Laboratories, Inc., Westbrook, ME, USA) as recommended by the manufacturers. MAP detection was assessed by processing two homogenates formed with mucosa from ileocecal valve (ICV) and distal ileum (DI) in one and jejunal caudal lymph node (JC-LN) in the other. Isolation in duplicate home-made Herrold and Löwenstein-Jensen media containing 2 mg/L of mycobactin J (Allied Monitor, Fayette, MO, USA) was carried out by inoculation of an aliquot, in accordance with Juste et al. [29]. At the same time another aliquot of the same homogenates was used for amplification of specific MAP IS*900* DNA with a commercially combined DNA extraction, purification, and real-time PCR kit (Adiapure-Adiavet; Adiagene, Saint Brieuc, France) as indicated by Vázquez et al. [23, 25, 28]. Typical histological PTB lesions were investigated in three tissue sections (ICV-DI, JC-LN, and ileal LN), which were fixed in 10% neutral-buffered formalin, dehydrated, embedded in paraffin, cut at 4 *μ*m sections, and stained with hematoxylin-eosin (HE). If granulomatous lesions consistent with PTB in HE-stained sections were observed, an additional section was stained with the Ziehl-Neelsen method for acid-fast bacilli. Histopathological lesions were classified into* focal*,* multifocal*,* diffuse lymphoplasmacytic*,* diffuse intermediate,* and* diffuse histiocytic* types, according to González et al. [22] and Vazquez et al. [23]. These four diagnostic protocols have been described in detail elsewhere [23, 25, 28].

ELISA was the least sensitive diagnostic method if referred to MAP isolation (%Se = 38.8 (95% CI = 30.9–46.7)) or histopathology (%Se = 17.2 (95% CI = 13.3–21.1)) and showed good specificity values (over 97%) for both reference tests. This resulted in moderate and poor agreement with tissue culture (kappa = 0.452) and histopathology (kappa = 0.156), respectively. On the contrary, rtPCR was a more sensitive test if compared with the same references (tissue culture: %Se = 79.6 (95% CI = 73.1–86.1); histopathology: %Se = 41.8 (95% CI = 36.8–46.9)), showing moderate agreement with tissue culture (kappa = 0.456) but poor agreement with histopathology (kappa = 0.177). Additionally, histopathology appeared as a more sensitive reference method for PTB diagnosis (%Se = 77.6 (95% CI = 70.8–84.3)) than tissue culture (%Se = 31.1 (95% CI = 26.4–35.9)) when they were evaluated together. On average, fair agreement was estimated for these two methods (kappa = 0.237).

Based on these phenotypical results, animals were further grouped into three PTB forms:* apparently free*,* latent *PTB, and* patent* PTB [25]. In total 366 PTB cases were considered of which 294 were* latent*, and the remaining 72 were* patent* ones. The number of* apparently free* animals (without lesions) accounted to 406 animals.

### 2.3. SNP Selection and Genotyping Process

Twenty-four SNPs in* NOD2 *(rs109601360, rs43710288, rs43710289, and rs43710290),* SLC11A1 *(rs109453173, rs110090506),* SP110* (rs136859213, rs133080973, and rs110480812),* TLR2 *(rs110491977, rs68268259, rs41830060, rs109971269, rs41830058, rs43706434, and rs43706433),* TLR4 *(rs29017188, rs43578097, and rs43578100), and* CD209 *(rs208222804, rs209491136, rs211654540, rs208814257, and rs210748127) bovine genes, previously analyzed in MAP infection association studies, were selected for this study [5–10].

Genomic DNA was extracted and purified from whole blood samples using the commercial QIAamp Mini Kit (Qiagen, Hilden, Germany) and following manufacturer's instructions. Genotyping of selected SNPs was carried out by TaqMan OpenArray technology (Life Technologies, Carlsbad, USA) and subsequent allele assignation was carried out using Autocaller v1.1 software (Life Technologies, Carlsbad, USA). Each genotyping array included duplicated negative and positive controls. To guarantee the genotyping quality, three control parameters were checked using PLINK v. 1.07 statistical software [30]: sample call rate (≥80%), SNP call rate (≥80%), and Hardy-Weinberg equilibrium (HWE). Consequently, 136 animals (67* apparently free*, 59* latent* PTB, and 10* patent *PTB) were removed from the statistical analysis because of their low-call rate. The remaining 636 animals were represented as follows: 339* apparently free*, 235* latent* PTB, and 62* patent *PTB (Table 1). All SNPs satisfied the HWE in the three subgroups (*P* > 0.05 after FDR adjustment) [31]. Minor allele frequency (MAF) of the 24 SNPs is shown in Supplementary Table 1 in Supplementary Material available online at http://dx.doi.org/10.1155/2014/321327.

### 2.4. Statistical Analysis

Analysis of associations between individual SNPs and PTB forms (*latent* versus* apparently free, patent *PTB versus* apparently free,* and* latent *PTB versus* patent *PTB) was performed with the WGassociation function from SNPassoc package in R2.14.0 software [32, 33], which offers the possibility of testing the minor alleles from each genetic marker under five different genetic models simultaneously, codominant, dominant, recessive, overdominant, and log-additive, and adjusting each model by age data. For all statistic tests the *P* values were corrected to avoid false positives (type I error) by permutation procedures (1,000,000 permutations) [34]. Odds ratio (OR) and their 95% confidence intervals (CI) for significant genotype-phenotype associations were also calculated with the same software.

Evaluation of PTB diagnostic methods in relation to the two methods considered as reference (tissue culture and histopathology) was done using the WinEpi software (http://www.winepi.net/) in terms of sensitivity (Se) and specificity (Sp) with their corresponding 95% confidence intervals (CI). Cohen's kappa statistic was also calculated to evaluate the agreement between tests. This statistic was interpreted as follows: <0.2 poor, 0.2–0.4 fair, 0.4–0.6 moderate, 0.6–0.8 good, and >0.8 excellent.

## 3. Results

As shown in Table 2, up to nine polymorphisms in* SLC11A1* (1),* SP110* (1),* TLR2* (2), and* CD209* (5) genes were found to be preliminarily associated with the* latent* PTB form when compared with the* apparently free* subgroup. However, the association was only confirmed for the rs208222804 SNP in* CD209 *gene under the log-additive model (*P* < 0.0034 after permutation procedure) (Table 2). According to this model, animals carrying the minor allele (C) in the rs208222804 SNP were less likely to develop* latent *PTB than those without it (OR = 0.64, 95% CI = 0.48–0.86).

In the* patent *PTB versus* apparently free* analysis, three genotypic associations involving the* NOD2* and* SP110* genes were detected but they did not reach the statistical level of significance after corrections under any of the models (Table 3). No association of the* latent *and* patent *forms with the panel of 24 SNPs in the six candidate genes was found (Table 4).

## 4. Discussion

This study represents the first approach to assess the association between the newly defined* latent *and* patent *forms of paratuberculosis (PTB) and a set of selected polymorphisms, therefore indirectly evaluating the hypothesis that some SNPs in innate immunity-associated genes may affect the type of granulomatous lesions in paratuberculosis dairy cattle.

During the early periods of MAP infection as well as in its* latent* forms, it is well known that infection can go unnoticed because immunopathological changes or bacterial loads do not reach enough magnitude as to cause clinical signs or yield positive results in the current laboratory diagnostic methods. As many researches point out, this major drawback needs to be strongly considered when designing genetic association studies and defining the basis to classify individuals [16, 19, 21, 24]. Herein, in order to achieve a high diagnostic sensitivity level, in addition to* in vivo* serological testing, tissue samples were examined by three diagnostic tests: culture, rtPCR, and histological examination. In fact, the latter has been found to be more sensitive than any of the other methods [23, 25] and pivotal for grading bovine PTB [25]. As a consequence, it appeared appropriate to replace the traditional phenotypical definition of cases by the newly defined PTB forms while using the* apparently free* animals as the control group.

Although bovine PTB is widely accepted to be a multigenically regulated infectious disease [16–21, 35], in a similar manner as it is presupposed to occur in its human multifactorial inflammatory bowel disorder counterpart—Crohn disease—[36] and in accordance with what is assumed for the pathogenesis of the major pathogenic mycobacterial agent in humans (*Mycobacterium tuberculosis*), only one genetic association between* latent *PTB and* CD209 gene* was confirmed (Table 2). The results from the log-additive model suggested that the minor allele (C) in the rs208222804 SNP was associated with a reduced likelihood of occurrence of the* focal* granulomas that are characteristic of the* latent* form, in relationship with the* apparently free* group (*P* < 0.0034 after permutation procedure; OR = 0.64, 95% CI = 0.48–0.86). This result is also in agreement with reported observations on the protective nature of allelic variants in two polymorphisms in* CD209* promoter region (rs4804803 and rs735239) against human tuberculosis [37, 38]. Even though protection against a form of infection might always be desirable, this SNP association alone would not provide a relevant effect on PTB control because open disease and high MAP shedding levels would only occur in the* patent* PTB. On the contrary, the* latent* form seems to be associated with some type of resilience as these animals seem to be slaughtered at older ages (data not shown) probably because farmers with a PTB problem would keep those animals that can withstand a heavily infected environment without developing clinical disease longer.

It is also somewhat noteworthy that none of the analyzed SNPs in the six candidate genes were found to be associated with* patent *PTB form (Tables 3 and 4). Since the characterization of this PTB form is most closely related to those forms of infection which include shedding MAP in feces and testing positive to the ELISA test [25], one could assume a relative similarity to those animals defined as cases in our previous studies [5–8] and therefore expect to confirm those already associated SNPs. In this sense, several causes could explain the lack of positive association results on this occasion. As mentioned above, our preliminary case-control association studies aimed to identify genetic variations related to microbiological and/or immunological evidences of infection [5–8], while, in this paper, histopathological observation was used for determining the phenotypical categories. Additionally, in those previous works phenotypical classification strongly leaned on classifying as uninfected controls only those animals that had tested negative in microbiological and immunological diagnostic methods. On the contrary, in the current study the* apparently free* subgroup represents a state with much lower chances of having hidden or minimal MAP infection lesions. Nevertheless, and that may be more decisive, it is important to underline that a few proportions of animals showing* patent *PTB (*n* = 62) were considered in the association analysis compared to the large number of those with* latent* PTB, almost four times higher (*n* = 235). For this reason, the effect of studied SNPs could be somewhat confounded. Thus, the observation of some nearly significant associations suggests that the involved SNPs could actually have some degree of association that would show up only in a larger sample of cattle. Our study shows that investigating genetic factors in a subset of animals according to their* in vivo* tests' results can draw genetic associations only to the progressive forms of PTB infections. These tests' efficacy, as seen in Table 1, might be reasonably well supported in terms of sensitivity and specificity relative to histopathology, if* latent* forms are not taken into account. Indeed, there were up to five SNPs in* NOD2, SP110, SLC11A1,* and* TLR2* genes that showed preliminary associations when comparing cattle showing* patent *PTB with those without lesions or with* latent *PTB (Tables 3 and 4). Interestingly, two SNPs in* SP110* gene (rs136859213 and rs110480812) were identified in both comparisons under diverse genetic models (Tables 3 and 4), which may point out to an effect in PTB progression in agreement with a trend in the same sense observed in human tuberculosis [39], though this tendency remains somewhat controversial [40]. Loss of statistical significance after permutation procedures, however, makes impossible to draw any solid conclusion and would require further investigation.

Consequently, the genetic associations shown here should be understood as an approximation to lead future researches. In this sense, since it was observed that there was a small effect on MAP infection when considering each SNP individually, then the use of models for interactive effects could shed light on specific multigenic contributions of these SNPs to the development of PTB forms.

## 5. Conclusion

Our study provided a new approach and one novel phenotypic-genotypic association for better understanding pathological features occurring in bovine PTB. Although no firm conclusions can be drawn to fully explain the genetic component of the diverse lesion patterns caused by MAP, especially for* patent* PTB, because of the limited sample size, preliminary data suggest that rs208222804 SNP in* CD209* gene is associated with the development of* latent* PTB form. Further studies considering interactive effects of SNPs in these candidate genes are needed to confirm these findings.

## Supplementary Material

Minor allele frequency (MAF) of studied SNPs in *NOD2, SLC11A1, SP110, TLR2, TLR4*, and *CD209* bovine genes is available in Supplementary Table 1

## Figures and Tables

**Table 1:** Characterization of paratuberculosis (PTB) status of the subgroup of 636 animals considered in genotypic association analysis, according to histopathological findings, seroprevalence estimates, and frequency of MAP detection in tissues.

	PTB status			Total
	*Apparently free *	*Latent *	*Patent *	
Histopathology	No lesions	*Focal* type	*Multifocal * and *diffuse* types	
ELISA				
(i) Number of negative (% specificity)	330 (97.4)	223 (—)	16 (—)	569
(ii) Number of positive (% sensitivity)	9 (—)	12 (5.1)	46 (74.2)	67
(iii) Mean OD ± SD	0.2 ± 0.2	0.2 ± 0.3	1.7 ± 1.0	2.1 ± 1.5
(iv) Percentage of ELISA positive	2.7	5.1	74.2	
(v) Histopathology complementary sensitivity (%)	—	1061.9	29.1	

Tissue culture				
(i) Number of negative (specificity)	310 (91.5)	192 (—)	9 (—)	511
(ii) Number of positive (sensitivity)	29 (—)	43 (18.3)	53 (85.5)	125
(iii) High bacterial load^1^ (*n*)	2	4	31	37
(iv) Percentage of culture positive	8.6	18.3	85.5	
(v) Histopathology complementary sensitivity (%)	—	266.7	11.0	

Tissue rtPCR				
(i) Number of negative (specificity)	254 (74.9)	164 (—)	4 (—)	422
(ii) Number of positive (sensitivity)	85 (—)	71 (30.2)	58 (93.5)	214
(iii) Mean Ct^2 ^± SD	36.2 ± 3.0	35.3 ± 3.5	25.4 ± 7.4	33.0 ± 7.4
(iv) Percentage of rtPCR positive	25.1	30.2	93.6	
(v) Histopathology complementary sensitivity (%)	—	105.1	2.8	

^1^High bacterial load was considered if >50 CFU/tube was observed. ^2^Mean threshold cycle (Ct) values are referred to the rtPCR positive subgroup. OD: optical density readings. SD: standard deviation.

**Table 2:** Association results from *apparently free* versus *latent* PTB logistic regression analysis.

Gene	SNP	Model	Genotypes	Percentage of a*pparently free *	Percentage of *latent* PTB	*P*
*CD209 *						
	rs208222804	Codominant	T/T; T/C; C/C	51.0; 41.8; 7.2	62.5; 34.1; 3.4	0.0096
		Dominant	T/T; T/C-C/C	51.0; 49.0	62.5; 37.5	0.0058
		Recessive	T/T-T/C; C/C	92.8; 7.2	96.6; 3.4	0.0471
		Log-additive	0, 1, 2	59.1	40.9	0.0024*
	rs209491136	Recessive	A/A-A/G; G/G	93.4; 6.6	97.0; 3.0	0.0469
	rs211654540	Codominant	A/A; A/G; G/G	51.8; 41.3; 6.9	61.8; 34.8; 3.4	0.0221
		Dominant	A/A; A/G-G/G	51.8; 48.2	61.8; 38.2	0.0152
		Log-additive	0, 1, 2	58.8	41.2	0.0063
	rs208814257	Dominant	C/C; C/G-G/G	49.8; 50.2	59.0; 41.0	0.0279
		Log-additive	0, 1, 2	58.9	41.1	0.0148
	rs210748127	Codominant	T/T; T/C; C/C	48.8; 42.5; 8.7	56.3; 40.3; 3.5	0.0158
		Recessive	T/T-T/C; C/C	91.3; 8.7	96.5; 3.5	0.0088
		Log-additive	0, 1, 2	59.0	41.0	0.0115

*SLC11A1 *						
	rs109453173	Overdominant	C/C-G/G; C/G	69.0; 31.0	60.4; 39.6	0.0348

*SP110 *						
	rs110480812	Codominant	A/A; A/G; G/G	54.8; 36.6; 8.6	45.9; 48.9; 5.2	0.0095
		Dominant	A/A; A/G-G/G	54.8; 45.2	45.9; 54.1	0.0356
		Overdominant	A/A-G/G; A/G	63.4; 36.6	51.1; 48.9	0.0035

*TLR2 *						
	rs109971269	Overdominant	T/T-C/C; C/T	74.3; 25.7	81.1; 18.9	0.0477
	rs43706433	Codominant	A/A; A/G; G/G	60.5; 34.4; 5.0	70.0; 25.2; 4.8	0.0484
		Dominant	A/A; A/G-G/G	60.5; 39.5	70.0; 30.0	0.0168
		Overdominant	A/A-G/G; A/G	65.6; 34.4	74.8; 25.2	0.0165
		Log-additive	0, 1, 2	59.4	40.6	0.0418

All models were adjusted for age at slaughter (years). SNPs and genetic models with a nominal *P* value <0.05 are only shown.

*Significant association (*P *< 0.0034 after permutation procedure). Odds ratio (OR): 0.64, 95% CI: 0.48–0.86. Threshold of significance for genetic models (after permutation procedure): *P*_codominant_ < 0.0027; *P*_dominant_ < 0.0039; *P*_recessive_ < 0.0024; *P*_overdominant_ < 0.0031; *P*_log-additive_ < 0.0034.

**Table 3:** Association results from *apparently free* versus *patent* PTB logistic regression analysis.

Gene	SNP	Model	Genotypes	Percentage of *apparently free *	Percentage of *patent* PTB	*P *
*NOD2 *						
	rs43710290	Codominant	C/C; T/C; T/T	73.8; 24.1; 2.1	61.3; 38.7; 0.0	0.0179
		Dominant	C/C; T/C-T/T	73.8; 26.2	61.3; 38.7	0.0345
		Overdominant	C/C-T/T; T/C	75.9; 24.1	61.3; 38.7	0.0135

*SP110 *						
	rs136859213	Dominant	C/C; C/T-T/T	86.6; 13.4	96.7; 3.3	0.0344
		Log-additive	0, 1, 2	84.6	15.4	0.0305
	rs110480812	Recessive	A/A-A/G; G/G	91.4; 8.6	82.3; 17.7	0.0467

All models were adjusted for age at slaughter (years). SNPs and genetic models with a nominal *P* value <0.05 are only shown.

No significant associations. Threshold of significance for genetic models (after permutation procedure): *P*_codominant_ < 0.0032; *P*_dominant_ < 0.0029; *P*_recessive_ < 0.0027; *P*_overdominant_ < 0.0024; *P*_log-additive_ < 0.0047.

**Table 4:** Association results from *latent *PTB versus *patent* PTB logistic regression analysis.

Gene	SNP	Model	Genotypes	Percentage of *latent* PTB	Percentage of *patent* PTB	*P *
*SLC11A1 *						
	rs109453173	Overdominant	C/C-G/G; C/G	60.4; 39.6	73.8; 26.2	0.0399

*SP110 *						
	rs136859213	Dominant	C/C; C/T-T/T	84.5; 15.5	96.7; 3.3	0.0237
		Overdominant	C/C-T/T; C/T	85.3; 14.7	96.7; 3.3	0.0341
		Log-additive	0, 1, 2	79.2	20.8	0.0215
	rs110480812	Codominant	A/A; A/G; G/G	45.9; 48.9; 5.2	45.2; 37.1; 17.7	0.0448
		Recessive	A/A-A/G; G/G	94.8; 5.2	82.3; 17.7	0.0233

*TLR2 *						
	rs109971269	Dominant	T/T; C/T-C/C	79.8; 20.2	69.4; 30.6	0.0194
		Overdominant	T/T-C/C; C/T	81.1; 18.9	71.0; 29.0	0.0171
		Log-additive	0, 1, 2	79.0	21.0	0.0357

All models were adjusted for age at slaughter (years). SNPs and genetic models with a nominal *P* value <0.05 are only shown.

No significant associations. Threshold of significance for genetic models (after permutation procedure): *P*_codominant_ < 0.0023; *P*_dominant_ < 0.0032; *P*_recessive_ < 0.0032; *P*_overdominant_ < 0.0038; *P*_log-additive_ < 0.0032.
