# Supplementary material for: Genetic Association Analysis of Paratuberculosis Forms in Holstein-Friesian Cattle
Source: Vet Med Int. 2014 May 20;2014:321327. doi: 10.1155/2014/321327 (PMC4055230; doi:10.1155/2014/321327)
Supplement: Supplementary file 1 — Minor allele frequency (MAF) of studied SNPs in NOD2, SLC11A1, SP110, TLR2, TLR4, and CD209 bovine genes is available in Supplementary Table 1 [file 321327.f1.pdf]

SUPPLEMENTARY TABLE 1: Minor allele frequency (MAF) of studied SNPs in *NOD2*, *SLC11A1*, *SP110*, *TLR2, TLR4* and *CD209* bovine genes (n=636).

**Gene**	**SNP**	**Minor Allele**	**Major Allele**	**MAF**
NOD2	rs109601360	A	G	0.2334
	rs43710288	T	A	0.4921
	rs43710289	A	C	0.4522
	rs43710290	T	C	0.1492
SLC11A1	rs109453173	G	C	0.2055
	rs110090506	T	A	0.1627
SP110	rs136859213	T	C	0.0700
	rs133080973	G	T	0.2537
	rs110480812	G	A	0.2887
TLR2	rs110491977	A	G	0.1270
	rs68268259	T	C	0.1292
	rs41830060	T	C	0.1193
	rs109971269	C	T	0.1302
	rs41830058	C	T	0.1751
	rs43706434	A	G	0.1268
	rs43706433	G	A	0.2040
TLR4	rs29017188	C	G	0.4199
	rs43578097	G	A	0.4199
	rs43578100	A	G	0.4114
CD209	rs208222804	C	T	0.2488
	rs209491136	G	A	0.2212
	rs211654540	G	A	0.2452
	rs208814257	G	C	0.2668
	rs210748127	C	T	0.2697
